# Predicting loss of independence among geriatric patients following gastrointestinal surgery

**DOI:** 10.1186/s13037-024-00424-w

**Published:** 2025-01-09

**Authors:** Michaela R. Cunningham, Christopher L. Cramer, Ruyun Jin, Florence E. Turrentine, Victor M. Zaydfudim

**Affiliations:** 1 Department of Surgery, University of Virginia, Charlottesville, Virginia USA; 2 Surgical Outcomes Research Center, University of Virginia, Charlottesville, Virginia USA; 3 Department of Public Health Sciences, University of Virginia, Charlottesville, Virginia USA; 4 Section of Gastrointestinal, Hepatobiliary and Pancreatic Surgery, Division of Surgical Oncology, Department of Surgery, University of Virginia, PO Box 800709, Charlottesville, VA 22908-0709 USA

## Abstract

**Background:**

While existing risk calculators focus on mortality and complications, elderly patients are concerned with how operations will affect their quality of life, especially their independence. We sought to develop a novel clinically relevant and easy-to-use score to predict elderly patients’ loss of independence after gastrointestinal surgery.

**Methods:**

This retrospective cohort study included patients age ≥ 65 years enrolled in the American College of Surgeons National Surgical Quality Improvement Program database and Geriatric Pilot Project who underwent pancreatic, colorectal, or hepatic surgery (January 1, 2014- December 31, 2018). Primary outcome was loss of independence – discharge to facility other than home and decline in functional status. Patients from 2014 to 2017 comprised the training data set. A logistic regression (LR) model was generated using variables with *p* < 0.2 from the univariable analysis. The six factors most predictive of the outcome composed the short LR model and scoring system. The scoring system was validated with data from 2018.

**Results:**

Of 6,510 operations, 841 patients (13%) lost independence. Training and validation datasets had 5,232 (80%) and 1,278 (20%) patients, respectively. The six most impactful factors in predicting loss of independence were age, preoperative mobility aid use, American Society of Anesthesiologists classification, preoperative albumin, non-elective surgery, and race (all OR > 1.83; *p* < 0.001). The odds ratio of each of these factors were used to create a sixteen-point scoring system. The scoring system demonstrated satisfactory discrimination and calibration across the training and validation datasets, with Receiver Operating Characteristic Area Under the Curve 0.78 in both and Hosmer-Lemeshow statistic of 0.16 and 0.34, respectively.

**Conclusions:**

This novel scoring system predicts loss of independence for geriatric patients after gastrointestinal operations. Using readily available variables, this tool can be applied in the urgent setting and can contribute to elderly patients and their family discussions related to loss of independence prior to high-risk gastrointestinal operations. The applicability of this scoring tool to additional surgical sub-specialties and external validation should be explored in future studies.

**Supplementary Information:**

The online version contains supplementary material available at 10.1186/s13037-024-00424-w.

## Background

Predicting postoperative outcomes for surgical patients remains a complex task, particularly among elderly patients [1, 2]. The tools currently available for preoperative risk stratification, such as the Risk Analysis Index (RAI) and the Modified Frailty Index (mFI), have primarily been validated for predicting mortality, complications, and readmission [3, 4]. While these metrics are important to clinicians and patients, patients and their families are also concerned about how an operation will potentially affect their quality of life, including their independence [5]. 

Existing studies have mixed success in predicting individual patients chance of loss of independence (LOI) after major abdominal surgery using these indices [6–9]. A modified version of the Edmonton Frailty Score has been validated to predict loss of independence after surgery; however, this scale has components that require completion of physical tasks, (e.g., Timed Up and Go test) [10, 11]. In the urgent or emergent setting, or in a brief clinical encounter, completion of these tasks may not always be feasible. Guidelines from the American College of Surgeons and the American Geriatrics Society advocate for a global assessment of elderly patients undergoing surgery, including screening for cognition, functional status, frailty, mobility, nutritional status, mental health including depression and substance use, risk of cardiac or pulmonary complications, and evaluation of the patient’s support network in addition to review of medical comorbidities and medications [12, 13]. This can often be time consuming in clinical practice, and may therefore not be performed during clinical encounters [14] or in the urgent or emergent setting (e.g., emergency department admissions). Therefore, establishing a screening tool that is fast and simple is of the utmost importance for facilitating frailty screening in clinical practice.

The objective of this study was to develop a novel predictive score that would stratify patients’ risk for loss of independence after an operation. The primary outcome measure was a composite outcome of discharge to facility that was not home, and a decrease in functional status from baseline to discharge. Additionally, we aimed to create a score based on variables that would be easy to collect, and would not require completion of physical tasks, that could therefore be applied to patients undergoing urgent or emergent operations. We hypothesized that a number of clinically relevant and easily accessible variables could predict loss of independence among the geriatric patient population selected for major gastrointestinal surgery.

## Methods

### Data and study population

This retrospective cohort study used American College of Surgeons National Surgical Quality Improvement Program (ACS- NSQIP) Geriatric Pilot Project Collaborative Geriatric Surgery Research File (GSRF) data available from January 1, 2014-December 31, 2018 (25 sites contributed 2014–2016 GSRF data and 20 sites contributed to 2017–2018 data). GSRF data were merged with ACS-NSQIP Participant Use Data Files (PUF). Patients were included if their age was greater than or equal to age 65, and if they underwent pancreatectomy, colectomy, proctectomy, or hepatectomy as defined by the Current Procedural Terminology (CPT) codes in Supplementary Table 1. Patients who had other surgical procedures were excluded as they were not systematically collected (e.g., small bowel or gastric) in ACS-NSQIP or were lower risk elective operations warranting individualized studies (e.g., bariatric). Patients were excluded from the analysis if they underwent outpatient surgery, were transferred from another hospital, were admitted from a facility other than home, from hospice, or from an unknown location. Patients were excluded if they experienced in-hospital mortality, left against medical advice, were discharged to hospice, or discharge status was unknown. Patients were additionally excluded if their functional status either before or after surgery was unknown. Supplementary Fig. 1 reports a modified CONSORT diagram demonstrating exclusion criteria for the patients in the study. The Institutional Review Board for Health Sciences Research has approved national de-identified ACS-NSQIP PUF and GSRF as Public Datasets at our institution.

### Variables

The ACS-NSQIP and GSRF abstracts demographic variables including age, sex, race, ethnicity, and Body Mass Index (BMI). Comorbid conditions collected include use of a mobility aid prior to surgery, fall within 1 year, weight loss prior to surgery, presence of dyspnea, history of chronic obstructive pulmonary disease (COPD), history of diabetes, smoking history, ventilator dependence, presence of ascites, hypertension requiring medications, presence of disseminated cancer, history of heart failure, history of renal failure, dialysis dependence, presence of preoperative wound infection, steroid use prior to surgery, history of bleeding disorders, preoperative transfusion requirement, presence of sepsis prior to surgery, American Society of Anesthesiologists (ASA) Physical Status Classification System score. Preoperative laboratory values collected include hematocrit, sodium, blood urea nitrogen (BUN), creatinine, white blood cell count, platelets, and albumin. Intraoperative and postoperative variables include non-elective surgery status and wound classification.

The primary outcome of interest was loss of independence, defined as a composite outcome of discharge to a facility and worsening of functional independence at discharge compared to admission. Discharge to a facility was defined as patients who were previously living at home alone, with others, or with support who were discharged to a multi-level senior community, rehabilitation, separate acute care, or a skilled or unskilled care facility. Loss of functional independence was defined by patients who had an independent functional status on admission, but were partially or totally dependent at discharge. Patients with loss of independence were compared to those who were discharged to home and had no loss of independence.

### Statistical analysis

Patients enrolled from January 1, 2014 through December 31, 2017 were used in the training dataset, while patients enrolled January 1 through December 31, 2018 were used in the validation dataset. Missing data was imputed as the median for the continuous variables or as the less risky category for the categorical variables.

Continuous variables were represented as mean ± standard deviation or median (interquartile range, IQR) and compared using the t-test or Wilcoxon rank-sum test, as appropriate. Categorical variables were represented as N (%) and compared using the chi-square test or Fisher’s exact test, as appropriate. ASA classes 1 and 2 were combined, and ASA classes 4 and 5 were combined for the analysis. Patient characteristics were compared between training and validation datasets, between patients with and without the outcome loss of independence.

The training dataset was used to generate the logistic regression (LR) model and scoring system to predict the outcome. Continuous variables were evaluated by the Restricted Cubic Spline Function to find the association between a single predictor to the response for a LR model. Variables with *p* < 0.2 in the univariable analysis when comparing those with and without postoperative loss of independence were used as candidate factors in the LR. The final full LR model was selected by Akaike Information Criterion (AIC) in a backward stepwise algorithm. Multicollinearity for the model was tested by the variance inflation factor (VIF). McFadden’s R-squared was calculated to select the six most important factors in the model. Based on the odds ratio (OR) from the simple LR model with the six selected factors, a scoring system (rounding the OR into an integer) was generated. The sum of the scores for the six factors are the risk score of the outcome for each patient. Then a one-factor LR model was conducted with the risk score as the only factor, to get the look-up table between the risk score and the predicted risk of loss of independence. The predicted risk by the full LR model, simple LR model, one-factor LR model, and the risk score were visually compared by scatter plot and boxplot. The discrimination and calibration ability of the scoring system was tested using the Area Under the Curve (AUC) of the Receiver Operating Characteristic (ROC) curve and the Hosmer-Lemeshow statistic in both training and validation datasets.

Statistical analyses were performed using R 4.2.2 software (R Foundation for Statistical Computing, Vienna, Austria) with the “Hmisc”, “pscl”, “car”, “pROC”, and “generalhoslem” packages.

## Results

A total of 6,510 patients were included in the study. The training dataset included 5,232 patients (80%), while the validation dataset had 1,278 patients (20%). A total of 841 patients (13%) lost independence after surgery. In the training dataset, there were 672 patients who lost independence (12.8%). In the validation dataset, there were 169 patients who lost independence (13.2%). Baseline characteristics for the training and validation datasets are shown in Table 1. Baseline comorbidities were similar between the training and validation sets, with the exception of race (*p* = 0.02), dyspnea (*p* = 0.01), age group (*p* < 0.001), and wound classification (*p* < 0.001). Median age was similar between the training and validation sets (72 vs. 73, *p* = 0.172).

**Table 1:** Baseline demographic characteristics and comorbid conditions in training and validation cohorts

Factors	Overall (*n* = 6510)	Training (*n* = 5232)	Validation (*n* = 1278)	*P*-value
Male Sex	2980 (45.8)	2404 (45.9)	576 (45.1)	0.572
Race				0.003
White Black or African American Asian American Indian or Alaska Native Native Hawaiian or Pacific Islander Unknown or Not Reported	5318 (81.7) 614 (9.4) 75 (1.2) 15 (0.2) 2 (0.0) 486 (7.5)	4266 (81.5) 472 (9.0) 62 (1.2) 13 (0.2) 1 (0.0) 418 (8.0)	1052 (82.3) 142 (11.1) 13 (1.0) 2 (0.2) 1 (0.1) 68 (5.3)	
Hispanic Ethnicity	246 (3.8)	207 (4.0)	39 (3.1)	0.128
Non-Elective Surgery	1244 (19.1)	991 (18.9)	253 (19.8)	0.486
Preoperative Use of a Mobility Aid	1082 (16.6)	854 (16.3)	228 (17.8)	0.191
Fall within the last year	557 (8.6)	453 (8.7)	104 (8.1)	0.551
Weight loss	360 (5.5)	285 (5.4)	75 (5.9)	0.555
Dyspnea				0.010
No With moderate exertion At rest	6019 (92.5) 460 (7.1) 31 (0.5)	4859 (92.9) 353 (6.7) 20 (0.4)	1160 (90.8) 107 (8.4) 11 (0.9)	
History of COPD	473 (7.3)	377 (7.2)	96 (7.5)	0.706
Insulin dependent diabetes	470 (7.2)	376 (7.2)	94 (7.4)	0.835
Current smoking within the last year	665 (10.2)	541 (10.3)	124 (9.7)	0.500
Ventilator use within 48 h prior to surgery	11 (0.2)	10 (0.2)	1 (0.1)	0.703
Ascites	31 (0.5)	25 (0.5)	6 (0.5)	0.969
Hypertension treated with medication	4212 (64.7)	3377 (64.5)	835 (65.3)	0.596
Disseminated cancer	624 (9.6)	506 (9.7)	118 (9.2)	0.633
History of Congestive Heart Failure	54 (0.8)	41 (0.8)	13 (1.0)	0.409
Renal Failure	14 (0.2)	10 (0.2)	4 (0.3)	0.496
Dialysis	31 (0.5)	23 (0.4)	8 (0.6)	0.386
Wound infection	100 (1.5)	80 (1.5)	20 (1.6)	0.925
Steroid use	365 (5.6)	292 (5.6)	73 (5.7)	0.855
History of bleeding disorder	287 (4.4)	229 (4.4)	58 (4.5)	0.801
Transfusion with 1 or more units of pRBCs prior to surgery	141 (2.2)	113 (2.2)	28 (2.2)	0.945
Sepsis	357 (5.5)	282 (5.4)	75 (5.9)	0.5
Wound Classification				< 0.001
Clean or Clean Contaminated Contaminated Dirty	5293 (81.3) 640 (9.8) 577 (8.9)	4299 (82.2) 509 (9.7) 424 (8.1)	994 (77.8) 131 (10.3) 153 (12.0)	
American Society of Anesthesiologists class				0.004
1 or 2 3 4 or 5	1795 (27.6) 4240 (65.1) 475 (7.3)	1487 (28.4) 3377 (64.5) 368 (7.0)	308 (24.1) 863 (67.5) 107 (8.4)	
Age group				< 0.001
65–69 70–74 75–79 80–84 85–89	2054 (31.6) 1864 (28.6) 1307 (20.1) 838 (12.9) 447 (6.9)	1699 (32.5) 1463 (28.0) 1020 (19.5) 699 (13.4) 351 (6.7)	355 (27.8) 401 (31.4) 287 (22.5) 139 (10.9) 96 (7.5)	
BMI group				0.899
Normal BMI Underweight Overweight Obese Morbidly Obese Extremely Obese	2052 (31.5) 166 (2.5) 2329 (35.8) 1243 (19.1) 476 (7.3) 244 (3.7)	1658 (31.7) 133 (2.5) 1869 (35.7) 986 (18.8) 388 (7.4) 198 (3.8)	394 (30.8) 33 (2.6) 460 (36.0) 257 (20.1) 88 (6.9) 46 (3.6)	
Preoperative HCT < 37	2557 (39.3)	2043 (39.0)	514 (40.2)	0.442
Preoperative sodium < 135	433 (6.7)	341 (6.5)	92 (7.2)	0.381
Preoperative BUN > 22	1194 (18.3)	950 (18.2)	244 (19.1)	0.439
Preoperative creatinine > 2	131 (2.0)	107 (2.0)	24 (1.9)	0.703
Preoperative WBC > 11	685 (10.5)	558 (10.7)	127 (9.9)	0.447
Preoperative albumin < 3.4	880 (13.5)	730 (14.0)	150 (11.7)	0.038

From the training dataset, 672 (12.8%) of patients lost independence after surgery. Table 2 compares the baseline demographic, comorbidity, and operative variables between patients who did and did not experience postoperative loss of independence in the training dataset. Patients who experienced loss of independence were significantly different in terms of sex, race, non-elective surgery, preoperative use of a mobility aid, fall within the last year, weight loss, dyspnea, history of COPD, insulin dependent diabetes mellitus, ventilator use prior to surgery, ascites, hypertension requiring medication, renal failure, dialysis dependence, wound infection, steroid use prior to surgery, bleeding disorder, transfusion requirement prior to surgery, preoperative sepsis, wound classification, ASA classification, age, BMI group, preoperative hematocrit, preoperative sodium, preoperative BUN, preoperative creatinine, preoperative white blood cell (WBC), and preoperative albumin (all *p* < 0.05). All variables with *p* < 0.2 in the univariable analysis were included as potential candidates in the LR model to predict loss of independence. The variables from the LR model were then selected backwards by AIC and tested for multicollinearity by VIF. The remaining variables form the full model, which included sex, Black or African American race, non-elective surgery, preoperative use of a mobility aid, fall within the last year, weight loss, insulin dependent diabetes, current smoking, ascites, wound infection, preoperative sepsis, wound classification contaminated, dirty, or infected, ASA class, age group, extreme BMI (< 18.5 or ≥ 40), preoperative BUN > 22, preoperative WBC > 11, and preoperative albumin < 3.4 (Table 3). No variables were found to be multicollinear.

**Table 2:** Baseline characteristics and comorbid conditions for patients who experienced loss of independence vs. no loss of independence in the training dataset

Factors	No LOI (*n* = 4560)	LOI (*n* = 672)	*P*-value
Male Sex	2142 (47.0)	262 (39.0)	< 0.001
Race			< 0.001
White Black or African American Asian American Indian or Alaska Native Native Hawaiian or Pacific Islander Unknown or Not Reported	3717 (81.5) 377 (8.3) 58 (1.3) 13 (0.3) 1 (0.0) 394 (8.6)	549 (81.7) 95 (14.1) 4 (0.6) 0 (0.0) 0 (0.0) 24 (3.6)	
Hispanic Ethnicity	184 (4.0)	23 (3.4)	0.447
Non-elective surgery	685 (15.0)	306 (45.5)	< 0.001
Preoperative use of a mobility aid	606 (13.3)	248 (36.9)	< 0.001
Fall within the last year	332 (7.3)	121 (18.0)	< 0.001
Weight loss	219 (4.8)	66 (9.8)	< 0.001
Dyspnea on exertion			< 0.001
No Moderate exertion At rest	4264 (93.5) 282 (6.2) 14 (0.3)	595 (88.5) 71 (10.6) 6 (0.9)	
History of COPD	304 (6.7)	73 (10.9)	< 0.001
Insulin Dependent Diabetes	302 (6.6)	74 (11.0)	< 0.001
Current smoking	462 (10.1)	79 (11.8)	0.197
Ventilator use within 48 h prior to surgery	5 (0.1)	5 (0.7)	< 0.001
Ascites	14 (0.3)	11 (1.6)	< 0.001
Hypertension treated with medication	2896 (63.5)	481 (71.6)	< 0.001
Disseminated Cancer	445 (9.8)	61 (9.1)	0.577
History of heart failure	34 (0.7)	7 (1.0)	0.416
Renal failure	4 (0.1)	6 (0.9)	0.001
Dialysis dependent	15 (0.3)	8 (1.2)	0.002
Wound infection	53 (1.2)	27 (4.0)	< 0.001
Preoperative steroid use	239 (5.2)	53 (7.9)	0.005
History of bleeding disorder	168 (3.7)	61 (9.1)	< 0.001
Transfusion with 1 or more units of pRBCs prior to surgery	79 (1.7)	34 (5.1)	< 0.001
Sepsis	155 (3.4)	127 (18.9)	< 0.001
Wound classification			< 0.001
Clean or clean contaminated Contaminated Dirty	3851 (84.5) 420 (9.2) 289 (6.3)	448 (66.7) 89 (13.2) 135 (20.1)	
American Society of Anesthesiologists class			< 0.001
1 or 2 3 4 or 5	1404 (30.8) 2902 (63.6) 254 (5.6)	83 (12.4) 475 (70.7) 114 (17.0)	
Categorical age			< 0.001
65–69 70–74 75–79 80–84 85–89	1600 (35.1) 1317 (28.9) 851 (18.7) 549 (12.0) 243 (5.3)	99 (14.7) 146 (21.7) 169 (25.1) 150 (22.3) 108 (16.1)	
BMI category			0.006
Normal Underweight Overweight Obese Morbidly obese Extremely obese	1430 (31.4) 110 (2.4) 1643 (36.0) 879 (19.3) 339 (7.4) 159 (3.5)	228 (33.9) 23 (3.4) 226 (33.6) 107 (15.9) 49 (7.3) 39 (5.8)	
Preoperative HCT < 37	1644 (36.1)	399 (59.4)	< 0.001
Preoperative sodium < 135	264 (5.8)	77 (11.5)	< 0.001
Preoperative BUN > 22	761 (16.7)	189 (28.1)	< 0.001
Preoperative creatinine > 2	70 (1.5)	37 (5.5)	< 0.001
Preoperative WBC > 11	396 (8.7)	162 (24.1)	< 0.001
Preoperative albumin < 3.4	502 (11.0)	228 (33.9)	< 0.001

**Table 3:** Logistic regression models to predict loss of independence

	Full Model		Short Model	
	OR (95% CI)	p-value	OR (95% CI)	p-value
Age, years				
65–69	Reference		Reference	
70–74	1.84 (1.38, 2.46)	< 0.001	1.69 (1.28, 2.23)	< 0.001
75–79	3.26 (2.45, 4.36)	< 0.001	2.75 (2.09, 3.63)	< 0.001
80–84	4.40 (3.24, 5.99)	< 0.001	3.37 (2.52, 4.51)	< 0.001
85–89	4.98 (3.50, 7.10)	< 0.001	3.82 (2.74, 5.34)	< 0.001
Preoperative use of a mobility aid	1.94 (1.57, 2.39)	< 0.001	2.28 (1.87, 2.77)	< 0.001
American Society of Anesthesiologists class				
1 or 2	Reference		Reference	
3	1.89 (1.47, 2.47)	< 0.001	1.91 (1.49, 2.48)	< 0.001
4 or 5	2.52 (1.76, 3.63)	< 0.001	2.88 (2.04, 4.07)	< 0.001
Preoperative albumin < 3.4	1.80 (1.43, 2.25)	< 0.001	2.21 (1.78, 2.74)	< 0.001
Non-elective surgery	1.76 (1.39, 2.21)	< 0.001	2.67 (2.19, 3.26)	< 0.001
Race: Black or African American	1.82 (1.38, 2.38)	< 0.001	1.70 (1.29, 2.21)	< 0.001
Wound Class				
Clean or clean contaminated	Reference			
Contaminated	1.42 (1.07, 1.88)	0.014		
Dirty/Infected	1.65 (1.20, 2.25)	0.002		
Sepsis	1.95 (1.34, 2.82)	< 0.001		
Weight loss	1.79 (1.28, 2.47)	< 0.001		
Fall within the last year	1.54 (1.17, 2.00)	0.002		
Male Sex	0.78 (0.65, 0.94)	0.009		
Insulin Dependent Diabetes	1.52 (1.11, 2.07)	0.008		
BMI < 18.5 or ≥ 40	1.49 (1.07, 2.06)	0.017		
Preoperative WBC > 11	1.36 (1.02, 1.78)	0.032		
Ascites	2.87 (1.11, 7.25)	0.026		
Current smoking	1.34 (0.99, 1.78)	0.051		
Wound infection	1.67 (0.95, 2.87)	0.067		
Preoperative BUN > 22	1.24 (0.99, 1.54)	0.053		

From the full LR model, McFadden’s R-squared was used to select the six most important variables for predicting the outcome: categorical age, preoperative use of a mobility aid, ASA class, preoperative albumin, non-elective surgery, and Black or African American race (all *p* < 0.001) (Table 3). These variables formed the short LR model. The odds ratio from the short LR model was rounded to an integer and used to create an integer score. These integer scores from the six risk factors were used to calculate the total risk score to predict loss of independence, as shown in Table 4. A one-score LR model, with the total risk score as the sole predictor, was used to predict the loss of independence. This model was employed to ascertain the relationship between the risk score and the predicted risk of the outcome, shown in Supplementary Table 2. Utilizing this simple score-based prediction tool involves calculating the total risk score for loss of independence by summing the scores in Table 4 and referencing the corresponding predicted risk in Supplementary Table 2.

**Table 4:** Score system to predict loss of independence

Factor	Description	Score
Age in years	65-69 70-74 75-79 80-84 > = 85	0 2 3 3 4
Preoperative use of a mobility aid	No Yes	0 2
American Society of Anesthesiologists class	1 or 2 3 4 or 5	0 2 3
Albumin (g/dL)	> 3.4 < 3.4	0 2
Surgery status	Elective Non-elective	0 3
Race	Other than Black or African American Black or African American	0 2

**Fig. 1:**
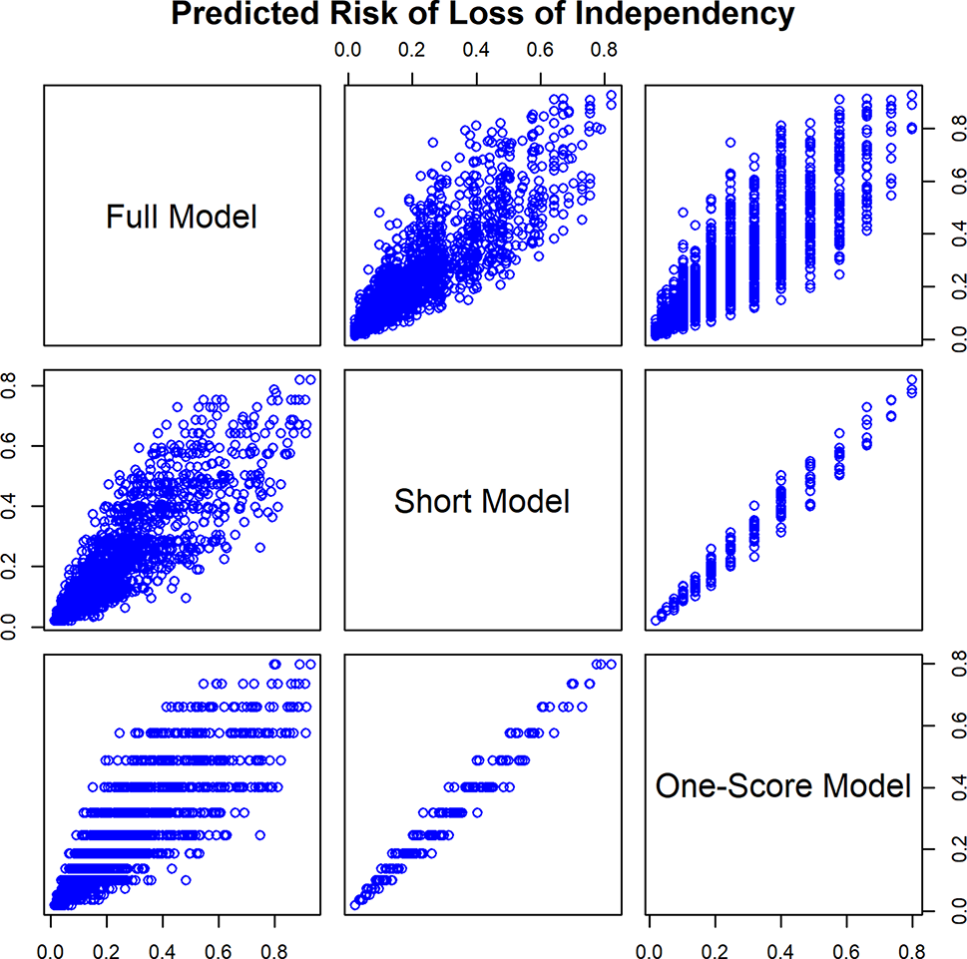
Scatterplot matrices compare the predicted risk of loss of independence by the three models. The predicted risk of loss of independence for each of the three models (the full logistic regression model, the short logistic regression model, and the one-score logistic regression model) are plotted against each other. The predicted risk of loss of independence for each of the models correlate well with each other

**Fig. 2:**
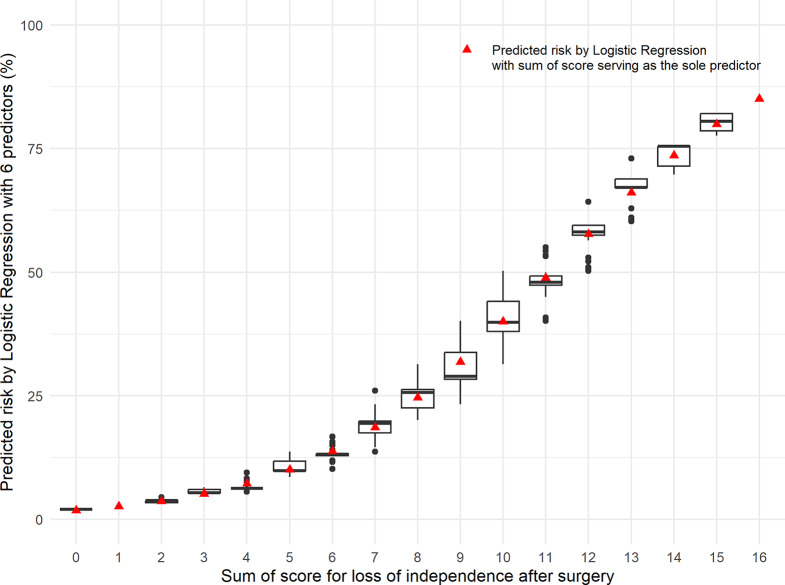
Box plot of the predicted risk of loss of independence from the short logistic regression model by the risk score. The box plots show the distribution of predicted risk of loss of independence from the short logistic regression model. The red triangles show the risk of loss of independence predicted by the one-factor model scoring system. The scores predicted by the score system correlate well with the median score predicted by the one-factor regression model

A scatterplot matrix comparing the predicted risk from the full LR model, the short LR model, and the one-factor LR model are shown in Fig. 1. The distribution of the predicted risk of loss of independence from the short model in each risk score group is shown in a box plot Fig. 2. The risk of loss of independence predicted by the risk score (Supplementary Table 2) is comparable to the median of the predicted risk by the six-factor short model.

**Fig. 3:**
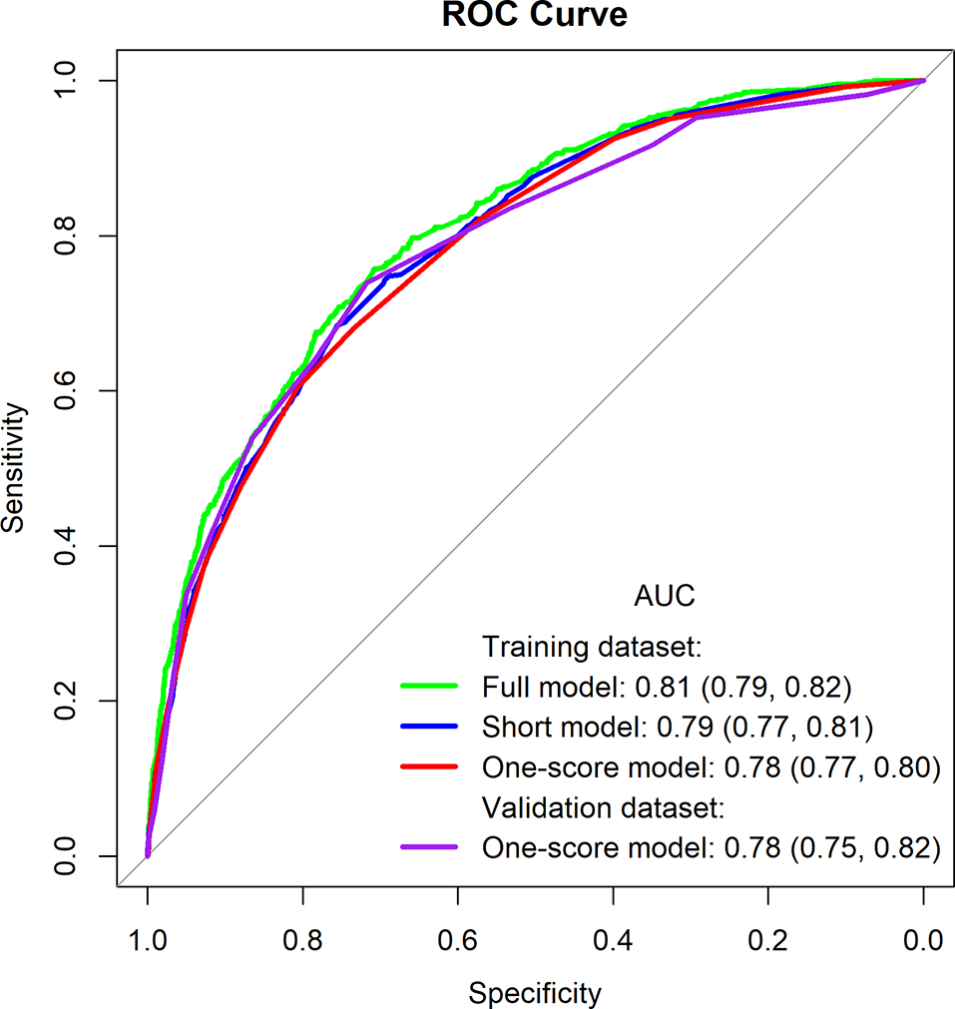
ROC Curves of the predicted risk of loss of independence for each of the models. The sensitivity and specificity of each of each of the models in the training dataset (the full logistic regression model, the short logistic regression model, and the one-score logistic regression model) and the sensitivity and specificity of the one-score logistic regression model for the validation dataset are shown. The models each have a comparable AUC (predicted AUC and 95% confidence intervals shown)

The models were evaluated by the AUC and Hosmer-Lemeshow test using the training and validation datasets. The ROC curves are shown in Fig. 3. The AUC in the training dataset for the full model, short model, and one-score model were 0.81 (95% CI 0.79, 0.82), 0.79 (0.77, 0.81), and 0.78 (0.77, 0.80), respectively. The AUC in the validation dataset for the one-score model was 0.78 (0.75, 0.82). The Hosmer-Lemeshow statistic for the one-score model in the training and validation dataset were 0.16 and 0.34, respectively.

## Discussion

In this study, we have created a risk calculator for loss of independence, defined by discharge to a destination other than home with a decrease in functional status after gastrointestinal surgery. The factors most predictive of postoperative loss of function were age, preoperative use of a mobility aid, ASA classification, albumin, non-elective surgery, and race. The created model reasonably predicted discharge to a destination other than home and decrease in functional status, with a ROC of 0.78 in both the training and validation datasets. The tool predicts inflection in LOI with a scores > 10. This score-based prediction tool demonstrates good discrimination and calibration capabilities.

We have previously described the use of geriatric-specific variables in predicting patient complications and discharge to facility after pancreatic operations among elderly patients [15]. We have also demonstrated that the RAI score is independently associated with patient mortality and failure to rescue during the 90 day post-operative period [16]. While mortality and major complications are important endpoints for both surgeons and patients, patients are also concerned about the way in which their lives will change after surgery, especially in regard to loss of independence. Combining a risk calculator validated to predict mortality or major complications, such as the RAI [3], and the risk calculator described in this study can potentiate informed consent process and patient decision-making prior to an operation.

Notably, we included patients undergoing both elective and non-elective operations in our risk calculator. These are very different clinical scenarios and patients in these groups should be counseled differently. Understanding that a non-elective operation incurs a significantly higher perioperative risk (of morbidity, mortality, and loss of independence), especially for patients at the extremes of physiologic and psychosocial conditions, may help the patient and family members make a better-informed decision about an urgent/emergent operation. The threshold of risk is different for every patient, and should be discussed preoperatively.

Loss of independence has been associated with an increased risk of readmission and mortality after discharge [17]. Discharge to a facility has also been associated with higher rates of six-month and one-year mortality [18, 19]. Identifying patients who are at risk of loss of independence and discharge to facility is critically important to improving their postoperative outcomes. Clinical factors known prior to the operation are critical in decision making. Whether or not a patient will have a major complication is unknown preoperatively and cannot be assumed during informed consent discussion. The factors contained in our model are six factors that are easy to establish during a pre-operative visit or informed consent discussion in an urgent setting. While some studies include similar factors such as age or ASA classification, others factors that have been associated with postoperative loss of independence include cognitive status, postoperative delirium, malignancy, Comprehensive Geriatric Assessment score, and Charlson Comorbidity Index score, income, type of insurance, and medical comorbid conditions [6, 18, 20, 21]. Our risk calculator independently incorporated several of these variables, while being simpler to implement but with similar discrimination capabilities, supporting future validation of its use in predicting discharge destination for elderly patients after gastrointestinal surgery.

While other frailty indices have been developed, they have limitations which make clinical applicability challenging. The Edmonton Frail Scale has been associated with discharge to a facility other than home in surgical patients greater than age 65^10^ and a modified Fried’s Frailty Index has correlated with lower functional independence one year after emergency abdominal surgery in elderly adults [22], however, both of these frailty indices do require an aspect of physical task completion, making them difficult to implement in the urgent or emergent setting, or during a brief clinical encounter. Our risk calculator has the advantage of containing only clinical variables, facilitating its use in time limited encounters. The self-reported domains on the Edmonton Frail Scale were recently shown to be predictive of discharge to a location other than home in surgical patients, making this somewhat easier to implement in clinical practice, although it is still limited by the self-reported nature of the questionnaire, and is subject to recall bias [23]. The Flemish version of the Triage Risk Screening Tool was also recently validated to predict short-term loss of independence in activities of daily living for elderly adults undergoing emergency abdominal surgery, however, this is also subject to significant recall bias due to the self-reported nature of the questionnaire [24]. As our risk calculator only includes objective data, it may prove advantageous to risk calculators relying on patient-reported data.

Understanding frailty and implementing clinical pathways may be one way to significantly improve outcomes and quality of life in elderly patients. In a cohort of frail patients who were admitted for emergency general surgery or trauma, implementation of a pathway focused on mobility and management of medications and comorbid conditions improved outcome measures for frail patients, including reduced length of stay, reduced readmission rates, and reduced loss of independence from 100–40%[25]. Among geriatric patients undergoing surgery, implementation of a geriatric-specific pathway significantly reduced total and direct costs, but especially for patients identified as frail [26]. We hope that our calculator can be one such tool to identify patients who are at risk for loss of independence, to promote informed decision making preoperatively, initiate inpatient and outpatient prehabilitation services, facilitate preoperative discharge and recovery planning, and improve clinical outcomes.

Although our scoring system has the advantage of being easy to use in time-limited settings, it faces the limitations of electronic medical record based screening tools [27]. The development of our screening tool is limited by the variables available in the ACS-NSQIP database, which cannot account for all the possible comorbidity or demographic data at the individual patient level, and cannot encompass the complexity of individual operations. Additionally, a number of patient socioeconomic factors such as family support, financial status, or patient’s level of education may influence clinical outcomes. These factors were not included in the dataset and as such could not be included in the analysis. The inclusion of race as a variable likely represents underlying systemic structures that have led to disparities in healthcare for patients of nonwhite race. Further research and advocacy may help to elucidate and eliminate these systemic biases and improve these patients’ outcomes. By design, the calculator also does not take into account a significant number of intraoperative or postoperative variables, many of which could be contributing factors to a patient’s postoperative discharge destination and loss of independence. This was intentional, however, as these factors would not be known preoperatively, and would not be available to be discussed when counseling a patient on the risk of loss of independence after surgery. However, the strength of our screening tool is potential for automation. Potential for automation has been recently advocated in shared surgical decision-making, and clinical management of geriatric patients by automation learning for older populations [28, 29]. 

In this study, we have created a risk calculator that can be used to predict loss of independence after gastrointestinal surgery. This scoring system uses variables readily available for geriatric patients, is easy to use during clinic discussion, and can be applied quickly in the urgent or emergent setting. This tool may be useful when discussing surgical risk, opportunities for prehabilitation, surgical outcomes, discharge and functional recuperation planning, prior to high-risk operations with elderly patients and their families. The applicability of the scoring tool to additional surgical sub-specialties as well as external validation should be explored in future studies.

## Electronic supplementary material

Below is the link to the electronic supplementary material.

Supplementary Material 1

## Data Availability

Data are available from American College of Surgeons National Surgical Quality Improvement Program Participant Use Data Files and American College of Surgeons Geriatric Pilot Project Collaborative Geriatric Surgery Research Files.

## Abbreviations

AIC: Akaike Information Criterion
ACS: NSQIP American College of Surgeons National Surgical Quality Improvement Program
ASA: American Society of Anesthesiologists
AUC: Area Under the Curve
BMI: Body Mass Index
BUN: Blood Urea Nitrogen
COPD: Chronic Obstructive Pulmonary Disease
CPT: Current Procedural Terminology
GSRF: Geriatric Pilot Project Collaborative Geriatric Surgery Research File
LOI: Loss Of Independence
mFI: modified Frailty Index
OR: Odds Ratio
PUF: Participant Use Data Files
ROC: Receiver Operating Characteristic
RAI: Risk Analysis Index
VIF: Variance Inflation factor
WBC: White Blood Cell
