# Supplementary material for: Predicting loss of independence among geriatric patients following gastrointestinal surgery
Source: Patient Saf Surg. 2025 Jan 9;19:1. doi: 10.1186/s13037-024-00424-w (PMC11715953; doi:10.1186/s13037-024-00424-w)
Supplement: Supplementary file 1 — Supplementary Material 1 [file 13037_2024_424_MOESM1_ESM.docx]

**Supplementary Figure 1: Modified CONSORT diagram demonstrating inclusion and exclusion criteria.**

|  |  |  |  |  |  |
| --- | --- | --- | --- | --- | --- |
| Patients age > 65 undergoing major abdominal operations in a hospital enrolled in ACS NSQIP and GSRF, 2014-2018,  Patients: 7776 | |  |  |  |  |
|  |  |  |  |  |  |
|  |  |  | *Outpatient n=25* | |  |
|  |  |  |  |  |  |
| 7751 | |  |  |  |  |
|  |  |  |  |  |  |
|  |  |  | *Transferred n=406* | |  |
|  |  |  |  |  |  |
| 7345 | |  |  |  |  |
|  |  |  |  |  |  |
|  |  |  | *Died in Hospital n=153* | |  |
|  |  |  |  |  |  |
| 7192 | |  |  |  |  |
|  |  |  |  |  |  |
|  |  |  | *Not Independent Preoperatively n=287* | |  |
|  |  |  |  |  |  |
| 6905 | |  |  |  |  |
|  |  |  |  |  |  |
|  |  |  | *Not Home Preoperatively  n=93* | |  |
|  |  |  |  |  |  |
| 6812 | |  |  |  |  |
|  |  |  |  |  |  |
|  |  |  | *Function Unknown Postoperatively  n=289* | |  |
|  |  |  |  |  |  |
| 6523 | |  |  |  |  |
|  |  |  |  |  |  |
|  |  |  | *AMA/Hospice/Unknown discharge location n=13* | |  |
|  |  |  |  |  |  |
| 6510 patients | |  |  |  |  |
|  |  |  |  |  |  |

**Supplementary Table 1: CPT codes of included cases**

| Operation | CPT codes |
| --- | --- |
| Pancreatectomy | 48120, 48140, 48145, 48146, 48148, 48150, 48152, 48153, 48154, 48155, 48999 |
| Colectomy | 44140, 44141, 44143, 44144, 44145, 44146, 44147, 44150, 44151, 44160, 44204, 44205, 44206, 44207, 44208, 44210 |
| Proctectomy | 44155, 44156, 44157, 44158, 44211, 44212, 45110, 45111, 45112, 45113, 45114, 45116, 45119, 45120, 45121, 45123, 45126, 45130, 45135, 45160, 45395, 45397, 45402, 45550 |
| Hepatectomy | 47120, 47122, 47125, 47130 |

**Supplementary Table 2: Look-up table to find the predicted risk of loss of independence by total risk score**

| Score | Risk of loss of independence |
| --- | --- |
| 0 | 0.018396 |
| 1 | 0.026089 |
| 2 | 0.036879 |
| 3 | 0.051894 |
| 4 | 0.072562 |
| 5 | 0.100588 |
| 6 | 0.137829 |
| 7 | 0.186007 |
| 8 | 0.246216 |
| 9 | 0.318293 |
| 10 | 0.400266 |
| 11 | 0.488231 |
| 12 | 0.576931 |
| 13 | 0.660934 |
| 14 | 0.735893 |
| 15 | 0.799312 |
| 16 | 0.850595 |
